# Predicting the methylation status of CpG islands from read distribution biases

**DOI:** 10.1186/s12864-025-12257-7

**Published:** 2025-10-30

**Authors:** Eldar T. Abdullaev, Dinesh A. Haridoss, Peter F. Arndt

**Affiliations:** 1 Department of Computational Molecular Biology, Max Planck Institute for Molecular Genetics, Berlin, Germany; 2 Department of Early Cancer Development and Prevention, Berlin Institute of Health (BIH) at Charité, Berlin, Deutschland; 3 Computational Health Center, Institute of AI for Health, Munich, Germany

**Keywords:** DNA methylation, DNA fragmentation, CpG islands

## Abstract

DNA methylation is an important epigenetic mark that plays a major role in transcriptional regulation, development and genome integrity. There are state-of-the-art methods, such as whole-genome bisulfite sequencing or long-read sequencing, which allow accurate detection of DNA methylation at single-base resolution. However, except for these specialized methods, information about DNA methylation status cannot be obtained directly from ordinary short-read sequencing data. Here we propose an approach to predict the methylation status from mapped read coordinates alone. It relies on previous findings that the DNA fragmentation process during library preparation is not random, but is affected by sequence context. In particular, DNA shearing leads to preferential hydrolysis of the sugar-phosphate backbone at CpG dinucleotides. Notably, methylated CpGs are approximately 30% more susceptible to fragmentation than unmethylated CpGs, likely due to subtle differences in the conformational dynamics. These differences are getting prominent when multiple NGS reads at CpG islands are analyzed. Our trained machine learning model is able to detect these biases and predict whether a CpG island of interest is methylated or not. We provide our methods as a tool, WGS2meth, that predicts CpG island methylation from whole-genome sequencing reads of individual or aggregated samples.

**Supplementary Information:**

The online version contains supplementary material available at 10.1186/s12864-025-12257-7.

## Background

DNA methylation, histone placement and their modifications are important epigenetic regulators that affect gene expression without modifying the nucleotide sequence. They play a critical role in transcriptional regulation, X-chromosome inactivation, spatial chromatin organization, inhibition of mobile genetic elements, DNA repair, etc. [1–3]. In mammalian genomes, DNA is typically methylated at the C5 position of a cytosine residue to form 5-methylcytosine (5mC). The vast majority of DNA methylation occurs in CpG dinucleotides and around $80\%$ of CpGs are methylated in a typical mammalian genomes [4–6]. 5mC is highly susceptible to spontaneous deamination to thymidine, thus vertebrate genomes are depleted with CpG dinucleotides [7–10]. In contrast to such a globally hypermethylated state there are so-called CpG islands (CGIs): CpG-rich DNA sequences of typical length in the range $500 - 2000$ bps [11]. CpG islands deserve special attention due to their unique genomic distribution and regulatory importance. In mammals, most CGIs are constitutively unmethylated. During the evolution of mammalian genomes, CGIs tend to vanish by *de novo* CpG methylation, which subsequently leads to increased rate of CpG to TpG transitions. On the other hand, purifying selection acting on functionally important CGIs could protect them from gradual CpG depletion and thus lead to their conservation [7, 12–14].

The CpG islands are often located in promoter regions of genes and play a regulatory role in gene expression. For instance, in vertebrate genomes approximately $70\%$ of all annotated gene promoters are associated with CGIs [15]. Typically, methylated CpG islands inhibit and unmethylated ones increase the expression of downstream genes. The distribution of DNA methylation marks varies significantly between tissues and developmental stages and depends on various factors affecting the cellular state, such as ageing, stress, activity of signaling pathways, and stochastic methylome changes [16–20].

Next-generation sequencing (NGS) has revolutionized methylation detection and many other genomics fields. Several experimental techniques have been developed to detect DNA methylation on a genome-wide scale at single-base resolution. Whole-genome bisulfite sequencing (WGBS) is the gold standard method for this task [21]. It involves treating DNA with bisulfite to convert unmethylated C into U (read as T during sequencing) while leaving 5mC intact which is subsequently read as C. Comparison of sequences from treated and untreated samples allows then the detection of 5mC bases. Alternatively, long-read sequencing platforms allow the detection of DNA covalent modifications including methylation while sequencing. Oxford Nanopore sequencing platform utilizes changes in electron current while DNA passes through a pore to distinguish covalent modifications of nucleotides [22, 23]. Pacific Biosciences SMRT achieves the same goal by tracking the kinetics of DNA polymerase as it replicates a DNA template [24]. However, despite these advances, most whole-genome sequencing data produced up to date comes from the short-read sequencing machines that can not distinguish modified from unmodified bases.

**Fig. 1:**
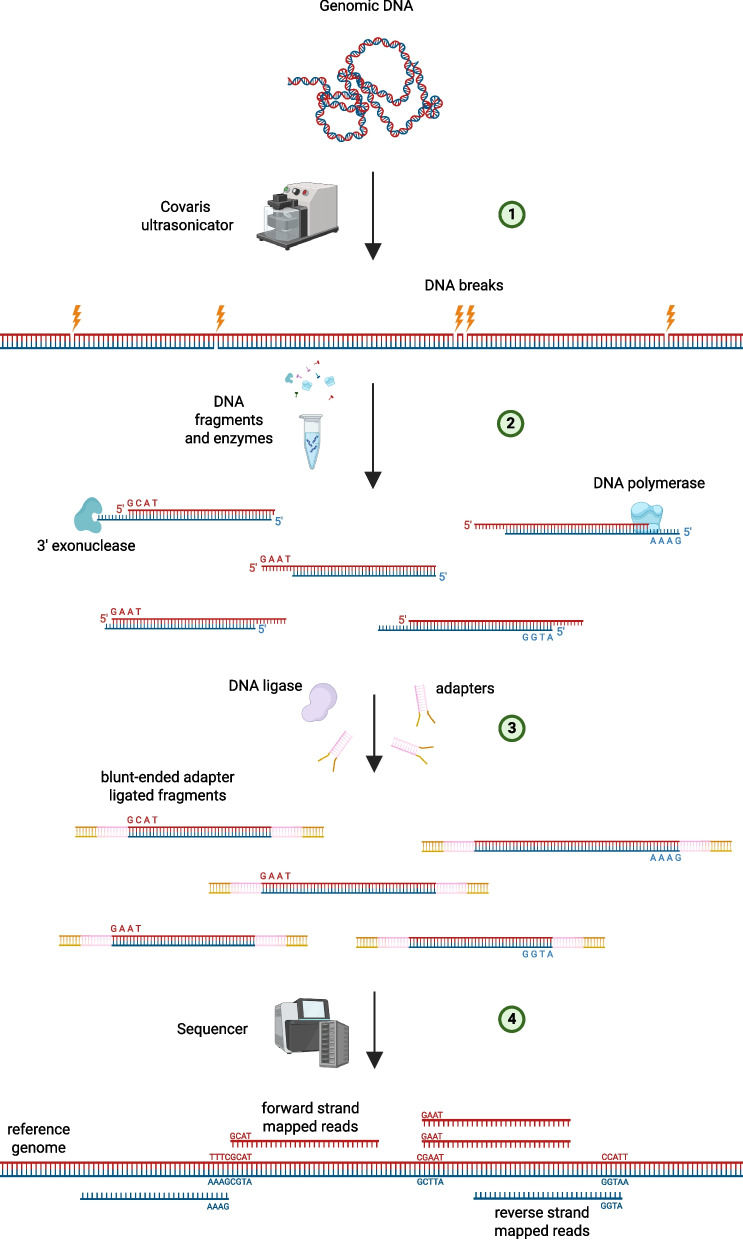
The scheme illustrates a general NGS pipeline. **(1)** Genomic DNA first has to be fragmented. Sugar-phosphate backbone hydrolysis of DNA molecules is stimulated by mechanic forces applied to it. **(2)** DNA overhangs (sticky ends) are converted to blunt by modifying 3’-ends by either prolonging with DNA polymerase or by shortening using 3’-end exonuclease. **(3)** Adapter sequences are ligated and DNA is amplified. **(4)** Finally, DNA fragments are sequenced and resulting reads are mapped on the reference genome. Read mapping positions inform us on the sites where genomic DNA was hydrolysed during the library preparation. On the figure only first four bases on 5’-ends of reads are listed and the corresponding sequences of the reference genome. Created with BioRender.com

Previously, it has been observed that NGS reads are non-randomly distributed across the genome and, for example, tend to be enriched in G/C-rich regions. Several scenarios have been proposed to explain these biases, ranging from the effect of PCR amplification to the mapping procedure [25–29]. In this study we focus on biases introduced by the non-uniform DNA fragmentation. The key insight is that the distribution of mapped reads along the genome reflects biases in the underlying DNA fragmentation process during the library preparation step (Fig. 1) [30, 31]. Genomic DNA is randomly sheared by physical forces (typically by sonication, nebulization or adaptive focused acoustics methods) into fragments which further proceed for sequencing [32]. Hydrolysis of DNA sugar-phosphate bonds is accelerated by mechanical forces and thus DNA fragmentation is a mechanochemical reaction by its nature [33]. Our method builds on earlier observations that the fragmentation of DNA happens non-randomly: some sequences are more “fragile” than others independently on the nature of a physical force applied. For example, sugar-phosphate backbone hydrolysis happens about 1.5 times more often in CpG dinucleotides than in other ones when DNA is affected by mechanochemical forces [31, 33]. Moreover, it has been observed that methylated CpG dinucleotides are more likely to be hydrolysed than unmethylated ones with the rate being about $30\%$ larger [30, 34]. This difference in the DNA fragmentation rates has been attributed to sequence-dependent conformational dynamics, likely modulated by the intensity of sugar ring *S* $\leftrightarrow$ *N* interconversion [33]. However, the precise biophysical mechanism is still not completely clear and has to be studied. Interestingly, the fragmentation rates for complementary dinucleotides are not identical. This can be explained by the fact that what appears as a double-strand break actually results from two separate single-strand breaks that occur at positions shifted relative to each other, generating sticky ends rather than a perfectly symmetrical break [31].

DNA fragmentation profiles are especially informative for the analysis of cell-free DNA (cfDNA). Fragmentation processes that lead to cfDNA formation are not random: they reflects chromatin state, nucleosome occupancy, activity of nearby transcription start site etc. [35–37]. Notably, methylated DNA exhibits a distinct fragmentation pattern: cfDNA fragments more frequently begin with CpG dinucleotides when those CpGs are methylated [38, 39]. This signal is sufficiently strong that a machine-learning model (XGBoost) trained on local coordinates of cfDNA fragments in an 11 bp window around a CpG dinucleotide, achieved AUC in the range: $0.827 - 0.959$ [40]. Similarly, a Hidden Markov Model (HMM) trained on fragment end positions accurately inferred methylation status across extended genomic elements, such as CpG islands [41]. It is important to note that cfDNA fragmentation differs from breaks introduced during standard NGS library preparation: cfDNA is generated by endogenous nucleases (*DFFB, DNASE1* and *DNASE1L3*) with specific site preferences [42]. The activity of *DNASE1L3*, in particular, is correlated with DNA methylation [43]. In contrast, the mechanical fragmentation of genomic DNA used in library preparation, produces a completely different end-motif spectrum. It lacks the CpG-start enrichment observed in cfDNA, while breaks are enriched between cytosine and guanine of CpG dinucleotides.

Here, we introduce a novel computational tool that makes predictions about DNA methylation status directly from ordinary whole-genome sequencing (WGS) reads. Based on the read coverage biases our tool can predicts whether CGIs are methylated or not on a genome-wide scale. We tested it on WGS samples from normal and cancerous cell lines and analyzed factors that can increase its accuracy.

## Results

Our goal was to train a classifier that predicts whether a CpG island (CGI) is methylated or not based on the distribution of mapped reads over the CGI body. During the library preparation step a genomic DNA is fragmented using mechanic forces. Mapping positions of resulting reads inform us about the sites where DNA breaks occurred. Specifically, we are focused on the fragmentation rates at the level of dinucleotides. For each read we extract its 5’-end coordinate and identify the dinucleotide formed by the read’s first base and its upstream neighbor in the reference genome. For example, if a read starts with the following bases 5’-*ACCGG...* and aligns to a region where *T* precedes it (i.e. *...TACCGG...*) we consider that genomic DNA was hydrolysed at a *TA* dinucleotide during library preparation. After collecting the information about affected dinucleotides we measure fragmentation rates as discussed later in this section. We analyze biases in read distribution by focusing on the 5’ coordinates of the mapped reads, since these coordinates define where DNA breaks happened during the fragmentation step (Fig. 1). One reason why we focus exclusively on 5’-ends is that 3’-ends are prolonged or shortened by T4 DNA polymerase during the library preparation step to produce blunt ends, while 5’-ends stay intact. Furthermore, 3’-end coordinates are normally trimmed to get rid of low quality bases and thus are not informative in our analysis.

We concentrate on methylation status of CGIs because they represent independent regulatory units with a discrete signal where most CGIs are either fully methylated or unmethylated (Sup. Fig. 1). We expect that for methylated CGIs, the distribution of reads is more biased towards start coordinates mapping inside of CpG dinucleotides (i.e. read 5’-end maps to the G base of CpG dinucleotides). Methylated CpG sites are especially susceptible to fragmentation during the library preparation phase prior to sequencing [30]. Mapping coordinate biases are identical for reads mapping to forward or reverse strands. We measured the following odds ratio to estimate relative fragmentation rates of dinucleotides in $i^{th}$ CpG island:

$$\begin{aligned} \rho ^i_{XY} = \frac{n^i_{XY}}{N^i} \big / \frac{m^i_{XY}}{L^i - 1} \end{aligned}$$

where *XY* is one of 16 possible dinucleotides, $n^i_{XY}$ - the number of reads starting at *XY* dinucleotides of $i^{th}$ CGI, $m^i_{XY}$ - the number of *XY* dinucleotides, $N^i = \sum _{x,y \in \{A,C,G,T\}} n^i_{xy}$ - the total number of reads whose 5’-end coordinates fall within the CGI of interest, $L^i$ - is the length of the CpG island. The $\rho ^i_{XY}$ values are measured for CGIs of human genome independently by counting reads mapping within the island of interest. Similarly $m^i_{XY}$ values are taken from the CpG island sequence. Under a uniform distribution of reads within a CGI, we would expect all $\rho ^i_{XY}$ values to be $\ \approx \ 1$. Any deviation from this value indicates a bias:

- $\rho ^i_{XY}> 1$ means reads start at the *XY* dinucleotides more often than expected by chance,
- $\rho ^i_{XY} < 1$ means they initiate less often than expected by chance.

**Fig. 2:**
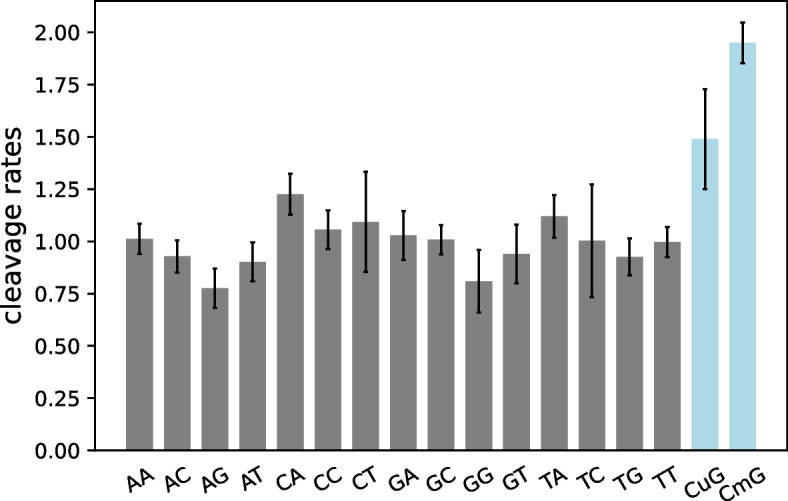
Relative fragmentation rates $\rho _{XY}$ for 16 unmodified dinucleotides and methylated CpGs. The $\rho _{XY}$ values plotted were computed using data from the entire genome, not limiting to a specific CpG island. In other words, all corresponding $n_{XY}$ and $m_{XY}$ values represent whole-genome measurements [30]. Error bars indicate the standard deviation of each $\rho _{XY}$ value. Methylation of CpGs results in a significant increase in fragmentation rate by $28\%$ in comparison with unmodified CpGs. This difference is utilized to distinguish methylated from unmethylated CpG islands. The $\rho$ values are taken from [30]

Fragmentation rates vary depending on the sequence involved and the DNA methylation status, as shown in the Fig. 2, where average fragmentation rates of dinucleotides are shown.

We trained a machine learning model which predicts the methylation state of CGIs from read 5’-end coordinates alone. To do that we calculated $\rho _{XY}$ values for all CGIs passing the filtering criteria, i.e. CGIs that can be assigned to either methylated or unmethylated state (see Methods for details). To generate a training dataset we downloaded WGS alignments from the 1000 Genomes project database and retrieved read coordinates from them [44]. For each sample we trained supervised machine learning models of the form:

$$\begin{aligned} y^i \ \ \sim \left[ \begin{array}{llll} \rho ^i_{AA}&\rho ^i_{AC}&...&\rho ^i_{TT} \end{array}\right] _{1 \times 16} \end{aligned}$$

where $y^i$ is a methylation state of $i^{th}$ CGI: $y^i \in \{0, 1\}$. In other words, we consider a two-class classification problem.

### Performance on lymphoblastoid cell lines and the effect of read coverage

**Fig. 3:**
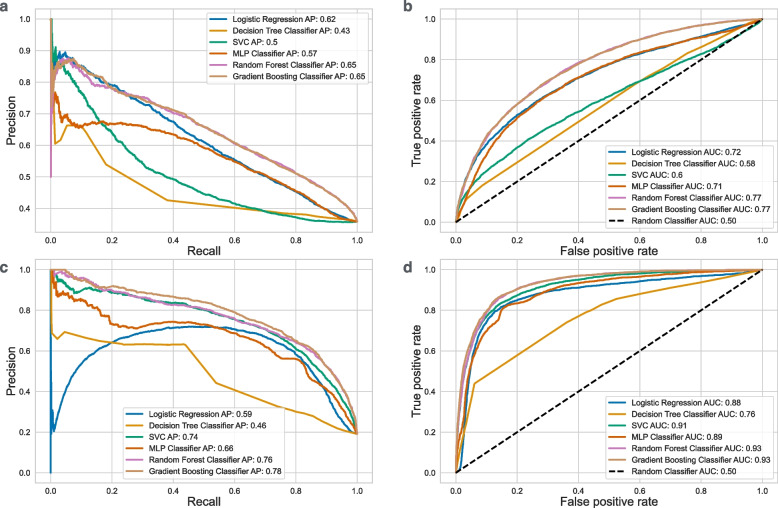
Precision-recall and receiver operator curves (ROC) measured for all machine learning models we used. The area under the curve (AUC) and the average precision (AP) values are given in the legend for each method. We used a single WGS sample from the 1000 Genomes project (**a** and **b**) and aggregated 20 samples (**c** and **d**) to train and test our models. The ensemble machine learning models, such as random forest and gradient boosting, perform the best

The lymphoblastoid cell line (LCL) samples were sequenced in the 1000 Genomes project, that is why we used whole-genome methylome of that cell line as a reference [44]. Among the models we tested, random forest and gradient boosting algorithms performed the best (Fig. 3). As our final model we selected the random forest which will be used in the rest of the text. Random forests is an ensemble learning method for classification or regression tasks that works by creating multiple decision trees during training. For classification tasks, the output of it is the class selected by a majority voting among trees. One can estimate the predicted probability of a certain class as a fraction of trees that vote for it.

**Fig. 4:**
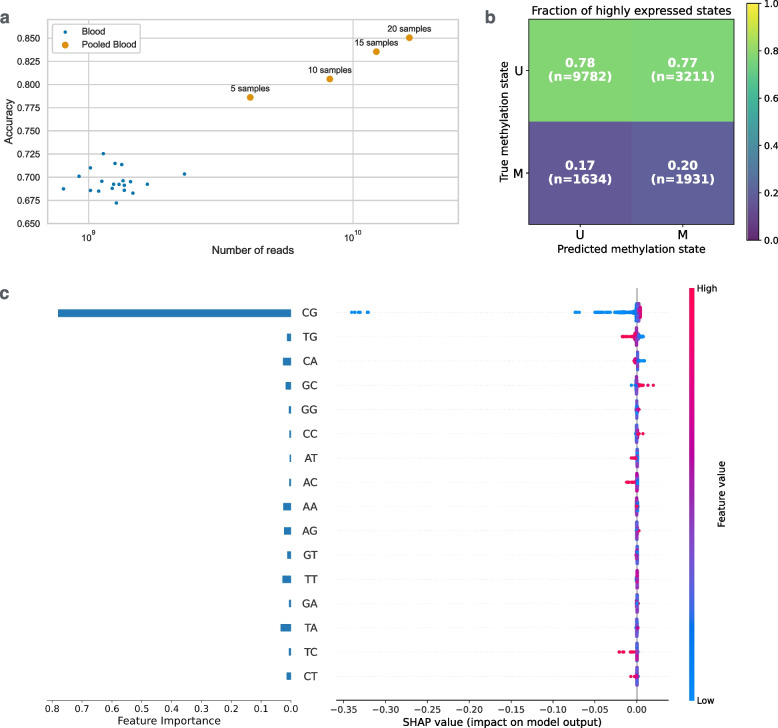
**a** Prediction accuracy of our tool on lymphoblastoid cell line datasets (referred as “Blood”) and their aggregates (“Pooled Blood”). Prediction accuracy increases with the total number of reads, because high coverage allows more accurate differentiation of read mapping biases in methylated and unmethylated CpG islands. **b** CpG islands are divided based on annotated and predicted methylation states. For each category the fraction of CGIs that overlap open chromatin regions is reported. **c** Feature importance of $\rho$ variables reported by the random forest algorithm is plotted on the left side. SHAP values of $\rho$ variables are on the right side. The fragmentation rates of CpG dinucleotides are the most informative in methylation status prediction

For high-coverage WGS samples we observe a range of accuracy values from 0.67 to 0.725 with the mean of 0.69 (Fig. 4a). Specifically, we used macro-averaged accuracy values: the average accuracy across the two classes (“methylated” and “unmethylated” CGIs). In general, the accuracy was higher for datasets with higher genomic read coverage. To further verify this, we aggregated reads from 5, 10, 15 and 20 samples belonging to the same cell type to create an artificial ultra-high coverage WGS dataset. The accuracy on the aggregated samples was even higher: 0.78, 0.81, 0.84 and 0.85 for 5, 10, 15 and 20 pooled samples, respectively. High read coverage provides better resolution of the model input data and leads to more accurate discrimination of read mapping biases in methylated and unmethylated islands. As an additional validation, we examined whether the predicted methylation statuses align with CpG island chromatin states. Using ChromHMM states associated with euchromatin and active transcription, we observed a clear enrichment of these active states among CGIs predicted as unmethylated (Fig. 4b). The difference was highly significant based on two-proportion Z-test: unmethylated (TN) versus methylated (TP) CGIs. By contrast, CGIs where our predictions disagreed with the reference annotation (FP and FN) showed no supporting chromatin signatures. Thus, the discordant calls are unlikely to reflect deficiencies of the annotation and most likely represent actual errors of our tool. Based on the feature importance reported by the trained random forest model, as expected, $\rho _{CG}$ is by far the most informative feature (Fig. 4c).

### Cancer cell lines and cross-training experiments

So far, we have analysed DNA from LCL cells of healthy individuals. However, we expect the same properties of DNA hydrolysis to be observed in any sample with CpG methylation. To prove this, we applied our method to WGS samples from cancer cell lines (listed in Methods) and compared our predictions with corresponding cell line methylomes [45, 46]. The prediction accuracy values were in a similar range to the ones observed for healthy blood samples: from 0.7 (*SKMEL30_SKIN* melanoma cell line) to 0.72 (*T84_LARGE_INTESTINE* colon adenocarcinoma cell line), with an average accuracy of 0.71. Overall, we can see consistency in the tool performance across tissue types and disease states.

**Fig. 5:**
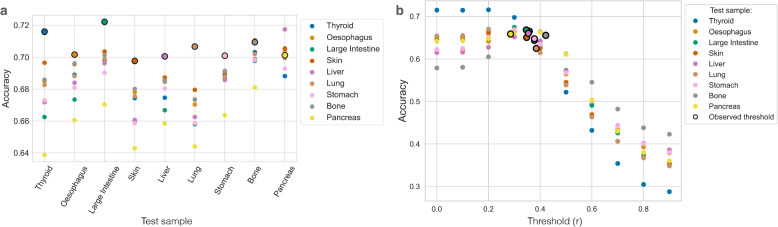
**a** Prediction accuracy values are plotted when a model is trained on one sample (indicated by colour) and tested on another (listed on the *X* axis). We use outlined circles to mark cases where training and test samples match. With the exception of the pancreatic adenocarcinoma (*DANG_PANCREAS*) and osteosarcoma (*U2OS_BONE*) cell lines, the best performance is reached when the model is trained on a matching sample. **b** The model is trained on the aggregated blood sample and tested on cancer cell line datasets. Different threshold values *r* are compared. The position where *r* is equal to the fraction of methylated CpG islands in the corresponding cell line is marked with the outlined circle. One can see that for all datasets except for *SW579_THYROID* the model reaches its peak performance in those positions

We pre-trained our model on data from one cancer cell line and applied it to another. These datasets belong to different tissues of origin and diseases, however, we expect that physical properties of DNA behave in the same way. The results of the cross-training experiments are shown at Fig. 5a. One can see that training on a different dataset does not lead to a substantial drop in performance, however, in most cases performance was slightly better when trained on the same cancer type. For some cases (pancreatic adenocarcinoma *DANG_PANCREAS* and osteosarcoma *U2OS_BONE* cell lines) the performance of the model trained on the same dataset was even lower than when the model was cross-trained on other samples. This is likely due to poor DNA methylation annotation in this particular cell line, heterogeneity of the cell population or copy number changes present in these cells. We also performed the same cross-training experiment, but this time used the model trained on the aggregated ultra-high coverage dataset (pooled 20 LCL samples). It performed well when tested on other cancer cell lines with the mean accuracy of 0.67. Technically, it means that our model can be trained once and applied on a broad set of whole-genome sequencing datasets without retraining. We implemented this approach in our WGS2meth tool.

### Performance across sequencing platforms and non-human species

**Fig. 6:**
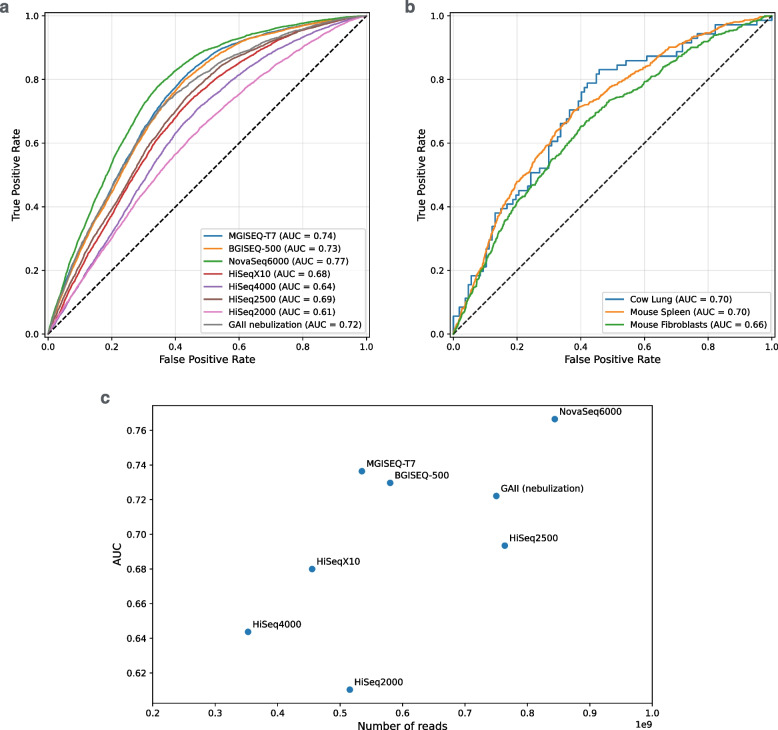
ROC curves across varying *r* values for samples stratified by sequencing platforms and library-preparation protocols (**a**) and by species (**b**). All datasets used Covaris ultrasound shearing, except “GAII” (Illumina Genome Analyzer II), which was prepared by nebulization, and “MGISEQ-T7” along with “BGISEQ-500”, which used enzymatic fragmentation. For each tested sample the area under the curve (AUC) value is given in the legend. Overall, the method performs consistently across platforms and generalizes to non-human species. One factor affecting its performance is coverage as illustrated at the (**c**) panel

To further demonstrate the universality of our method, we evaluated it on datasets from multiple short-read sequencing platforms drawn from a benchmark study [47]. Those include Illumina platforms (NovaSeq6000, HiSeq X10, HiSeq4000, HiSeq2500, HiSeq2000) and MGI ones (MGISEQ-T7 and BGISEQ-500). Additionally, we included Genome Analyzer II datasets from the phase 1 of the 1000 Genomes Project, which are the only samples prepared by nebulization rather than Covaris ultrasound shearing (all other Illumina samples) or enzymatic fragmentation (BGI/MGI samples) [48]. Additionally, samples from different species and tissues were also analyzed: mouse spleen and fibroblasts, and bovine lung samples [49–51]. Our default model, trained on aggregated blood samples, was applied to all human datasets. We trained species-specific models for each species in a way ensuring no data leakage between the training and test CGI sets (see Methods). Since the accuracy values could be misleading when tool is tested on unbalanced datasets with varying methylation fractions - we report the area under ROC curve (AUC-ROC) values for all runs (Fig. 6a,b). Overall, performance is consistent across platforms and fragmentation methods (mean AUC-ROC $\approx 0.70$) and generalizes to non-human species. Finally, Fig. 6c shows that read coverage exerts a stronger influence on performance than other technical aspects of the sequencing experiment. Interestingly, our method also performed well on WGS datasets where DNA is fragmented according to MGI library prep protocol, which utilizes fragmentation enzymes. Although no mechanical shearing is used - it seems that methylated CpG dinucleotides are the most affected by some types of enzymatic fragmentation. However, the scope and limitations of applying the method to enzymatically fragmented DNA require further evaluation.

### WGS2meth: implementation and usage

By default, one can run the WGS2meth tool on a WGS dataset of interest with the pre-trained model that is distributed with the code. Alternatively, one can train a new model on input dataset if corresponding methylome annotation is provided for training. This should lead to a better performance on samples produced using the same experimental protocol, data processing pipeline or belonging to the same tissue/species/condition. The only parameter that should be specified when using new samples is an expected fraction of methylated CGIs out of all CGIs in the genome (*r*). This allows to set a threshold on when to consider a CGI methylated. The random forest algorithm reports posterior probabilities of each CGI being methylated, predicted as the average vote over all decision trees in the ensemble (prior probabilities are equal $P(\text {meth.}) \ = \ P(\text {unmeth.}) \ = \ 0.5$, because the model was trained on a balanced dataset). CGIs are sorted based on the predicted probability of being methylated, reported by the random forest algorithm. The top *r* fraction of them is considered methylated, while CGIs from the rest $1 - r$ are unmethylated in the final output. The *r* parameter can either be inferred from a training dataset or explicitly given as an input. We studied the performance of the model when different *r* values were given as an input. The model was trained on the aggregated sample from the 1000 Genomes project and tested on the cancer cell lines (Fig. 5b) or trained and tested on non-matching cancer cell lines (Sup. Fig. 2). The highest prediction accuracy is observed when the expected fraction *r* is equal to a real proportion of methylated CGIs in the tested dataset.

## Discussion

Here we investigate whether we can predict DNA methylation from biases in read mapping coordinates. Our method is based on the previous observation that methylated CpGs are more prone to fragmentation when DNA is affected by mechanic forces of various nature. In practice, this effect becomes particularly prominent when analyzing NGS data, as non-uniform DNA fragmentation during library preparation results in the overrepresentation of reads starting within CpG dinucleotides (i.e., reads whose 5’-end maps to a guanine immediately preceded by a cytosine). Methylated CpG dinucleotides are more affected by hydrolysis at the library preparation step before sequencing than unmethylated ones. That is why the distribution of mapped reads over a CpG island can inform us on whether it is methylated or not. We utilize this principle to make predictions about a methylation state of CGIs using only one input: the coordinates of mapped NGS reads within a CpG island of interest. The accuracy of our tool was in 0.67 to 0.725 interval for the single samples we analysed, but there are factors that can improve it’s performance. For example, we found that the read coverage was a critical factor in prediction accuracy.

Our method relies on fundamental biophysical properties of DNA, such as it’s mechanic stability and it is proved to be universal when we tried it on samples from various human tissues and disease states, along with samples belonging to other species. It showed consistent performance across sequencing platforms (Illumina and MGI) and DNA fragmentation methods. In principle, it can be applied to any short-read sequencing dataset from organisms that exhibit CpG methylation. Given the huge amount of already produced short-read NGS datasets both from humans and other species, and a rather limited use of whole-genome bisulfite and long-read sequencing, our tool has the potential to close the gap between well-studied DNA sequences and corresponding unknown epigenetics. It can be especially helpful, when one has WGS data and no opportunity to do matching bisulfite sequencing experiments, for example, because of financial constraints or simply because one has no access to the original biological sample. In this case, our method provides the only way to infer methylome data. It is particularly useful for cohorts of samples of the same origin (same tissue/organism/cell type), as aggregation of reads from multiple samples markedly increases accuracy which, otherwise, may not be sufficient when applying WGS2meth on individual samples. Given the fact that WGS prices are constantly falling, new ultra-high coverage sequencing platforms are appearing and new technical advances are being made in the field ([52, 53]), we may expect higher coverage sequencing soon. This would lead to a huge increase in prediction accuracy according to our aggregation experiments. Unlike the described approach of aggregating reads “vertically” across multiple samples, we propose a less intuitive but theoretically justified “horizontal” aggregation of CGIs to improve predictive accuracy. If CGIs gain or lose methylation coordinately as functional groups, aggregating predictions across such CGI clusters could enhance the accuracy of group-level methylation state calls. For example, this approach can be applied for partially methylated domains (PMDs), where megabase-scale genomic regions exhibit consistent hyper- or hypomethylation. Such coordinated behaviour was observed in normal and more frequently in malignant cells [54, 55]. Considering CpG islands belonging to the same PMD as a group can increase prediction accuracy of the large-scale methylome changes. Also WGS2meth could extend for predicting methylation at other genomic elements, such as gene bodies.

We implemented this method in the WGS2meth tool which is simple in use: you only need the reference, CpG island coordinates and the bam file as your inputs. Overall, WGS2meth provides a cost-effective and retrospective avenue to obtain epigenetic insights from existing short-read WGS datasets, potentially closing the gap between genome sequencing and methylation profiling. The fact that such predictions are possible is quite surprising, as it bridges functionally unrelated data types by exploiting biases in one of them. It’s a good example of the “it’s not a bug, it’s a feature” approach implemented in practice.

## Conclusions

We trained a machine learning model capable of predicting DNA methylation from read mapping coordinates alone. Our method is based on the previous observation that methylated CpGs are more prone to fragmentation when DNA is subjected to mechanical forces, such as those applied during library preparation prior to sequencing. As a result, in WGS samples we observed more reads whose 5’-end coordinates map to CpG dinucleotides for methylated than unmethylated CpG islands. We used this bias to train a random forest classifier that can distinguish methylated from unmethylated CpG islands based on WGS read coordinates. We demonstrate the universality of our method, evaluate its performance across diverse datasets and discuss its limitations. It is publicly available as the WGS2meth tool.

## Methods

### Data

We used whole-genome sequencing (WGS) alignments from the phase 3 of the 1000 Genomes project. The following high-coverage samples were randomly picked: *HG00419, NA19625, NA19017, HG03052, NA18525, HG02568, HG03642, HG01112, HG01051, HG03742, HG00096, NA18939, HG00759, NA20502, HG00268, HG02922, HG01595, NA19238, HG01583* and *NA19648* [44]. The material used for sequencing originated from transformed lymphoblastoid cell lines (LCL) that were established from peripheral blood of healthy individuals. Thus as a reference methylome for the listed samples we used LCL methylome from the ENCODE project (ENCODE id: *ENCFF279HCL*) [56]. Other than that, we aggregated samples from the phase 1 of the 1000 Genomes project, where DNA was fragmented using nebulization (*HG00637, NA18628, NA19382, NA19401, NA19455*) [48].

We used WGS data of the cancer cell lines from the Cancer Cell Line Encyclopedia (CCLE) [45, 46]. The BAM files were downloaded from the SRA archive (SRA id: *SRP186687*), while matching methylomes were taken from the DepMap portal (https://depmap.org/portal). The cell lines that we used include: *DANG_PANCREAS, TE11_OESOPHAGUS, SHP77_LUNG, T84_LARGE_INTESTINE, SKMEL30_SKIN, SKHEP1_LIVER, NUGC3_STOMACH, U2OS_BONE* and *SW579_THYROID*. For all cancer cell lines, we specifically selected WGBS datasets that were derived from the same source material as the WGS data within the CCLE project. In these cases, both the sequencing data and the methylome annotations correspond not only to the same biological type, but also originate from the exact same biological source.

ChromHMM chromatin state annotations were downloaded from UCSC genome browser for the LCL (*GM12878*) [57]. Only those states associated with open or actively transcribed chromatin (*Active_TSS, Flanking_Active_TSS, Strong_transcription*) were collected out of all states and their overlap frequency with CpG islands was quantified.

Raw reads from the benchmark study where different sequencing platforms are compared were downloaded from SRA (BioProject id: *PRJNA600063*) [47]. There are Illumina platforms (NovaSeq6000, HiSeq X10, HiSeq4000, HiSeq2500, HiSeq2000) and MGI platforms (MGISEQ-T7 and BGISEQ-500) in the study. All samples were derived from human peripheral blood. As a reference we used whole-genome methylome from peripheral blood of healthy individual (GEO accession: *GSM848927*).

WGS datasets from healthy mouse were downloaded from [49] (SRA id: *SRR5224031*) and [50] (SRA ids: *SRR13341950, SRR13341952, SRR13341953, SRR13341954* and *SRR13341955*). In all cases only samples from healthy control mice were used. Reads were aligned to the GRCm38 (mm10) mouse reference genome. Samples belonging to the same tissue were aggregated. Reference methylome of mouse spleen was downloaded from ENCODE (*ENCSR662VFL*). Primary dermal fibroblasts methylome was taken from [58] study (GEO accession: *GSM5342492*). Healthy bovine lung datasets were taken from [51] (SRA ids: *SRR11235444* and *SRR11235445*) and aggregated. Reads were aligned to the bosTau9 bovine reference genome. Reference methylome was taken from the same study (GEO accession: *GSE147087*).

All CpG island coordinates and reference genomes were downloaded from the UCSC genome browser.

### Tools

We use samtools (1.21) to filter informative reads, so that only properly mapped pairs with no chimeric alignments, PCR or optical duplicates are left [59]. Raw reads are aligned with bwa (0.7.19-r1273). PCR duplicates are identified with gatk MarkDuplicates (4.6.2.0) command [60, 61]. We use bedtools (v2.30.0) to do all intersection and sequence extraction tasks [62]. Python package liftover (1.3.2) was used to transfer BED file coordinates between reference genomes builds. Packages scikit-learn (1.7) and shap (0.48.0) were used for the machine-learning tasks. All the routines needed to calculate $\rho ^i_{XY}$ values from the input BAM files are implemented in the preprocessing snakemake pipeline of the WGS2meth tool (Sup. Fig. 3) [63].

### Filtering criteria and data extraction

We call a CpG dinucleotide methylated or unmethylated if $>90\%$ of the WGBS reads agree on its status and it is covered by more than 10 reads. Alternatively, we assign “unknown” status to the CpG dinucleotide.

From all 27,081 CpG islands, we selected those with at least one CpG dinucleotide assigned to methylated or unmethylated status. If the number of methylated CpG dinucleotides was greater than the number of unmethylated CpG dinucleotides, we considered a CGI to be methylated and vice versa. Other than that, we filtered out those CGIs that overlapped segmental duplications, to reduce inaccuracies in methylation status assignment. Segmental duplication coordinates are downloaded from the UCSC genome browser (https://genome.ucsc.edu/). We ended up with about 14 thousand CGIs with known statuses, depending on the cell line analyzed (e.g. there are 14,061 CGIs, of which $19\%$ are methylated in the lymphoblastoid cell line).

For each CpG island *i*, we calculated odds ratios $\rho ^i_{XY}$ for 16 possible dinucleotides *XY* as described in the Results section. If all reads are randomly distributed across the genome, we would expect all values to be $\ \approx \ 1$. Any deviation from this means that reads start from *XY* dinucleotides more (if $\rho ^i_{XY}> 1$) or less (if $\rho ^i_{XY} < 1$) frequently than what is expected by chance. We only consider the 5’ coordinates of reads as a proxy for DNA break positions, as reads are often trimmed to a certain length at their 3’-ends. All $\rho$ values are measured inside of each CGI independently. If a dinucleotide *XY* is not covered by read starting positions or is absent in a CGI sequence, we set $\rho _{XY} = 0$.

### Machine learning algorithms

We evaluated several machine learning classifiers, including naive Bayes, logistic regression, decision tree, support vector machine (SVM), multilayer perceptron with multiple hidden layers (500, 200, 100, 1) and a sigmoid activation function, random forest, and gradient boosting. To optimize each model, we applied a grid search technique to identify the best hyperparameters of each model. Because most CGIs are unmethylated in the human genome, the training data are class-imbalanced. We solved this by undersampling the majority (unmethylated) class in the training set. Depending on the analysis, the test fold was either left at its natural class distribution - when evaluating the effect of *r* on accuracy or testing WGS2meth across platforms and species - or subsampled to balance the classes in all other cases. Whenever the model was trained and evaluated on the same sample (e.g., species-specific non-human models), we used 10-fold cross-validation and reported accuracy/AUC values on the held-out folds. Otherwise, the trained model was evaluated on the entire test dataset. The best-performing random forest model, trained on the aggregated 1000 Genomes Project samples, is implemented in our WGS2meth tool.

We used two criteria to show feature importance for predicting methylation status. The Random Forest importance is measured as the Mean Decrease in Impurity (MDI). For a given feature, it is computed by summing up the Gini impurity reductions at every node where the feature is used to split and weighting each reduction by the number of samples reaching that node. The final importance score is the average of this values across all trees in the ensemble. SHAP (SHapley Additive exPlanations) values represent the contribution of each feature to a specific prediction, showing how much a feature pushed the prediction value higher (positive) or lower (negative SHAP value) compared to the average prediction [64].

When the model was trained on one sample and applied to another, we did not perform batch correction to demonstrate the tool’s universality. Specifically, during the training step, read coordinates from a single sample were collected, and for each CGI, the $\rho ^i_{XY}$ values were calculated. The random forest model was then trained to predict the annotated methylation state of CGIs. This model was subsequently tested on read coordinates from other alignments derived from distinct biological tissues. The results demonstrate that the mechanochemical properties of DNA are universal, meaning a model trained on one sample can be readily applied to another, regardless of the biological source.

## Supplementary Information

Supplementary Material 1.

## Data Availability

All the scripts we used can be found in the following repository: https://github.com/Dinesh-Adhithya-H/MethylationAnalysis. All input BAM files, methylome annotations and CpG islands coordinates are available in open access (see Methods). The WGS2meth tool can be downloaded here: https://github.com/Dinesh-Adhithya-H/MethylationPrediction.

## Abbreviations

NGS: Next-generation sequencing
CGI: CpG island
WGS: Whole-genome sequencing
WGBS: Whole-genome bisulfite sequencing
cfDNA: Cell-free DNA
LCL: Lymphoblastoid cell line
PMD: Partially methylated domain
