# Supplementary material for: Predicting the methylation status of CpG islands from read distribution biases
Source: BMC Genomics. 2025 Oct 30;26:973. doi: 10.1186/s12864-025-12257-7 (PMC12574136; doi:10.1186/s12864-025-12257-7)
Supplement: Supplementary file 1 — Supplementary Material 1. [file 12864_2025_12257_MOESM1_ESM.pdf]

Supplementary figures

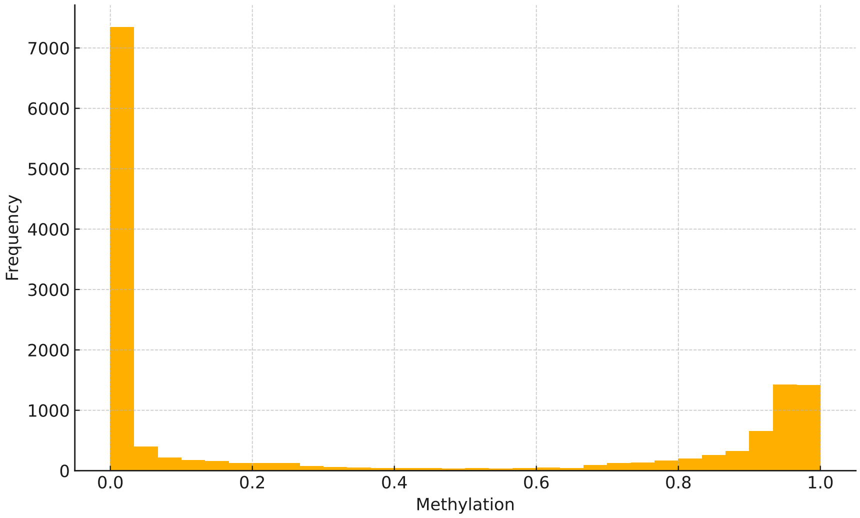

Sup. Fig. 1: The histogram of methylation levels among CpG islands of the pancreatic adenocarcinoma cell line (*DANG_PANCREAS*). The Y axis shows the number of CpG islands in each methylation bin.

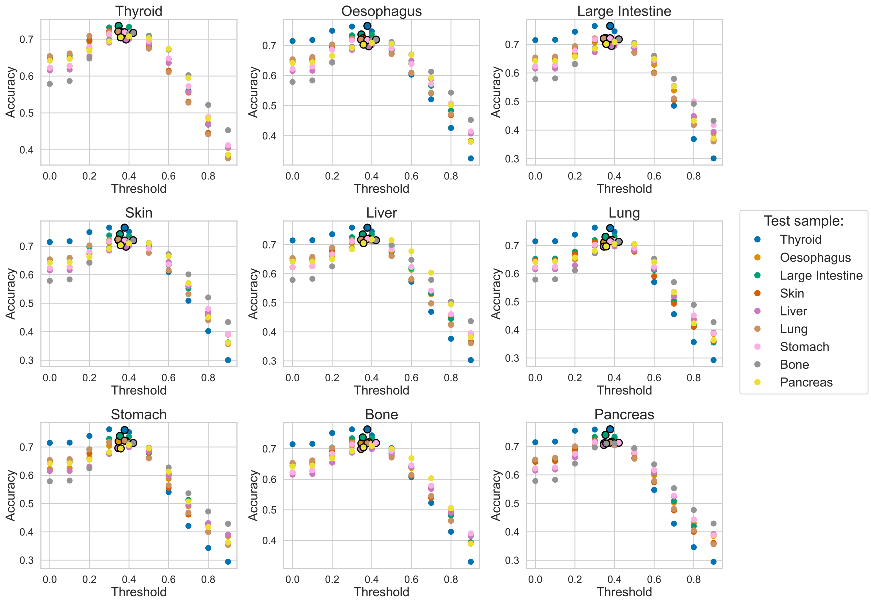

Sup. Fig. 2: The model is trained on one cancer cell line (listed in the title) and tested on the other cancer cell line datasets (excluding the one used for training). Different threshold values r are compared. The position where r is equal to the fraction of methylated CpG islands in the corresponding cell line is marked with the outlined circle. The model reaches its peak performance in those positions.

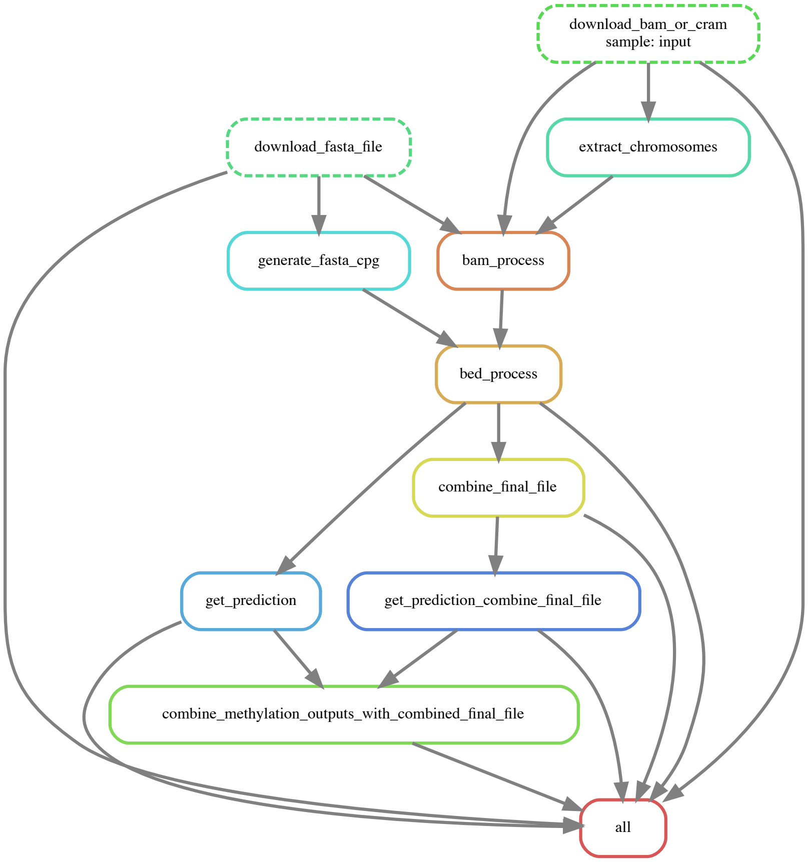

Sup. Fig. 3: The scheme of the snakemake pipeline implemented in the WGS2meth tool (prediction mode).
